# Expression, prognosis and functional role of Thsd7a in esophageal squamous cell carcinoma of Kazakh patients, Xinjiang

**DOI:** 10.18632/oncotarget.16966

**Published:** 2017-04-08

**Authors:** Zhichao Hou, Abulajiang Abudureheman, Lei Wang, Ayshamgul Hasim, Julaiti Ainiwaer, Haiping Zhang, Madiniyat Niyaz, Halmurat Upur, Ilyar Sheyhidin

**Affiliations:** ^1^ Department of Thoracic Surgery, First Affiliated Hospital of Xinjiang Medical University, Urumqi, Xinjiang Uyghur Autonomous Region, PR China; ^2^ Department of Pathology of Medical University of Xinjiang, Urumqi, Xinjiang Uyghur Autonomous Region, PR China; ^3^ Clinical Medical Research Institute, First Affiliated Hospital of Xinjiang Medical University, Urumqi, Xinjiang Uyghur Autonomous Region, PR China; ^4^ Department of Uyghur Medicine, Xinjiang Medical University, Urumqi, PR China

**Keywords:** esophageal squamous cell carcinoma, Thsd7a, Kazakh patients, prognosis, function

## Abstract

Thsd7a (Thrombospondin type 1 domain containing 7a) is a critical transmembrane protein. Studies have indicated that Thsd7a was associated with cytoskeletal organization, cell migration and filopodia formation. However, the involvement of Thsd7a remains elusive in human Esophageal Squamous Cell Carcinoma (ESCC). Consequently, immunohistochemistry and reverse transcription-polymerase chain reaction were utilized to study the correlation between the expression of Thsd7a and clinical-pathological characteristics. The influence of Thsd7a on apoptosis, cell proliferating activity, cell cycle, migratory and invasive capacity was determined in Eca 109 and EC 9706 cell lines *in vitro*. And the influence on proliferating activity was testified using naked mice model *in vivo*. In addition, the potential molecular mechanism was tested by microarray. It was discovered that there is a certain correlation between Thsd7a and the Kazakh ESCC. By knocking out Thsd7a, the invasion, migration and proliferation could be decreased. And it could also arrest the cell cycle at G1 phase and increase the apoptosis rate. It was further verified that Thsd7a had obvious effect on proliferation in naked mice with xenograft of Eca109 cells. Finally, it was uncovered by microarray analysis that a variety of tumor genes and pathways related to Thsd7a. Together, it was demonstrated that Thsd7a might have a certain degree of carcinogenesis in ESCC.

## INTRODUCTION

As a malignant tumor of poor prognosis, esophageal cancer shows significantly regional and national differences in the world [1, 2]. Xinjiang is known as one of the regions where esophageal cancer incidence is among the highest ones in China, particularly Kazakh incidence (68.88/100 thousand) [3]. However, the molecular mechanism influencing growth, invasion, and metastasis is still unclear for esophageal cancer of Kazakh patients.

Thsd7a (Thrombospondin type 1 domain containing 7a) gene is located in 7p22, which is a vulnerable region of multiple chromosome abnormalities of the chromosome [4]. Thsd7a encodes thrombin sensitive protein 1 7a domain, which is a transmembrane protein [5]. The full-length of Thsd7a is 260kDA, which is divided into three parts: the membrane anchoring zone 17KDA, functional area 210KDA, amino terminal 33 KDA [4, 5].

Thsd7a was firstly found in the human brain cDNA library by Nomura in 1994, originally named as KIAA0960 [6, 7]. At present, the research of Thsd7a is mainly focused on Osteoporosis, Membranous Nephropathy and Obesity [8–12]. In 2008, Mori et al, reported that the TSP1 region of Thsd7a was associated with the generation and activation of TGF-β [8, 13]. And Chi EM found that Thsd7a had 6 WSX rich sequences, which could bind and stimulate TGF-β activation [14]. In animal experiments, Du J illustrated that the repeated O fucose glycosylation of the Thsd7a family was able to significantly limit TGF-β induced epithelial mesenchymal transition (EMT) [15]. And Meng Wei et al, confirmed that the WSXW sequence of Thsd7a could make FAK phosphorylation [16], which promoted cell proliferation and inhibited cell apoptosis through ERK1/2 and P38 of MAPK pathway (RAS-RAF- ERK). Moreover, Meng Wei et al, found that Thsd7a contained more than 10 TSRs and RGD sequences [16]. While TSRs could affect endothelial cell migration and angiogenesis [17]. TSRs and RGD promoted endothelial cell viability, through identi- fying and combining the paxillin and αvβ3 integrin (αvβ3 integrin, receptor for TGF-β1/β3 to mediate cell adhesion function) [5, 18, 19]; and they enhanced the capacity of cell infiltration and movement [20]. Based on previous non-tumor researches, supplementary data 1 is the structure and combined target sketch map of Thsd7a. In addition, chieh and Meng proposed that the expression of Thsd7a was collocated with platelet endothelial cell adhesion molecule-1 (PECAM-1) in placental villous and umbilical vein for Human endothelial cells [5, 16]. They supported that Thsd7a was a potential target for anti-angiogenesis therapy [5, 16]. And authors suggested that Thsd7a might have a function as tumor suppressor gene, which was likely to facilitate the progress of tumor growth without it [5]. These studies suggested that Thsd7a might be related to cell adhesion, growth, differentiation, proliferation and apoptosis, which are likely to relate with the occurrence and development of tumor. However, the expressing state of Thsd7a in cancerous people as well as the effect on developing activity of cancer was still unrevealed.

In the present study, the expressing state of Thsd7a in ESCC of Kazakh patients was first assessed. It was revealed by results that the expressing level of Thsd7a was elevated in the progressing process of tumors, which indicated that Thsd7a was positively related to Clinical stage and differentiation. We then inhibited Thsd7a expression in ECa 109 and EC 9706 cells using small interfering RNA (siRNA) and the result uncovered that the knocking down of Thsd7a damaged the process of cell proliferating, reduced migratory and invasive ability, resulted in block of Cell Cycle, and increased apoptosis rate in Eca 109 and EC 9706 cells. We also tried to verify the Thsd7a's role in tumorigenesis and development by BALB/c Nude mice model in bodys. In addition, it was uncovered by microarray analyses that multiple pathways essential to cancerous occurrence and development were mediated by the knocking down of Thsd7a, which provided a valuable insight into the principle of Thsd7a mediating ESCC occurrence and progression. In summary, it was intensively suggested that Thsd7a was causally related to ESCC progression, which implicated that Thsd7a could act as a possible target for diagnosis and treatment of ESCC.

## MATERIALS AND METHODS

### Ethics statement

Informed consents were obtained from all patients and the protocol of this study was approved by the ethics committee of the First Affiliated Hospital of Xinjiang Medical University.

### Samples of patients

Esophageal tissue samples were gathered from inpatient or outpatient Kazakh patients with ESCC who came from the Thoracic Surgery Department in First Affiliated Hospital of Xinjiang Medical University. The stages of cancer tissues for Kazakh patients were determined according to the criteria proposed by American Joint Committee on Cancer classification. Tissue samples (*n* = 117) acquired from Pathology department were fixed with formalin and embedded in paraffin (FFPE). Freshly frozen esophageal tissues or FFPE samples were gathered during the surgical procedure. FFPE specimens or fresh-frozen esophageal tissues were gathered within 30 min after being resected. None of these patients were given radiotherapy or chemotherapy before operation. Tumors and adjacent normal tissues were evaluated by an experienced pathologist and immediately frozen with liquid nitrogen and preserved under the temperature of −80°C. The samples were stained with eosin and haematoxylin to verify the diagnosis and evaluate tumorous cellular content, metastasis as well as pathological grade. Tumorous cells without necrosis constituted seven tenth of tumorous samples in total.

The grading of cancers was inclusive of 5 AJCC stage 0-I, 89 AJCC stage II, and 23 AJCC stage III. The classification based on differentiation included 26 high level cases, 64 moderate level cases as well as 27 low level cases. Forty-one patients exhibited metastasis in lymph nodes. The median age of esophageal cancer patients was 60 years (ranged from 38-82 years).

### Immunohistochemistry (IHC)

IHC staining was conducted using anti-human Thsd7a rabbit polyclonal antibody (1:100, Atlas antibody). Tissue blocks embedded in paraffin were sliced into sections with the thickness of 3mm. Subsequently xylene and alcohol accompanied with distilled water were used to dewax and rehydrate the samples respectively. Then the samples were heated in citrate buffer (pH 6.0) under the temperature of 95°C for fifteen minutes to retrieve antigen. Three percent of hydrogen peroxide was utilized to incubate samples after which were chilled down to ambient temperature with the aim of blocking the activity of peroxidase. Subsequently the samples were incubated under the temperature of 4°C with first antibody and washed 3 times using phosphate-buffered saline (PBS), biotin-labeled secondary antibody (ZSGB-BIO, Beijing, China) was added for 15 min. After being washed with PBS, samples were counterstained with haematoxylin and diaminobenzidine.

Blind tests were conducted by 2 experienced pathologists separately who came to agreement on numbers of all tumorous samples to evaluate the intensity and ratio of stained tumorous cells. Cytoplasm and cell membrane Thsd7a were determined quantitatively in accordance to staining percentages (0%-100%) and intensity values (0, 1+, 2+, or 3+). Percentage (ranged from 0 to 300) and intensity values were multiplied to calculate IHC expressing scores. Positively stained Thsd7a was defined as (> 0); weak positive (0-100); moderate positive (100-200); and strong positive (200-300).

### Cell culture

Human ESCC Eca109 and EC 9706 cell lines were bought from WuHan University (Wu Han, China) and both of which were cultured using RPMI (Roswell Park Memorial Institute) 1640 added with 10% FCS(fetal calf/ bovine serum) as well as 1% penicillin/ streptomycin in a 5% Carbon dioxide incubation incubator under the temperature of 37°C.

### Construction and cell transfections

Eca 109 and EC 9706 cell lines were inoculated into 6 well plates and grown to 60%-80% confluence. The eukaryotic expressing vectors containing full-length complementary DNA (cDNA) fused with Flag Tag in the carboxyl terminal were bought from Invitrogen (SHANGHAI, CN); Thsd7a inhibitor (siRNA 1/siRNA 2: 10620318-278129 C07/10620318-278129 C09), and the scrambled sequence (siRNA 1/siRNA 2: CAGAACAGA CAAGA GAACAAAUAUU/UCCCUGUGAAGAGC CUGCCUGUUAU) were synthesized by Invitrogen (SHANGHAI, CN). Lipo- fectamine RNAi-MAX (Invitrogen, CA, USA) as well as Opti-MEMI (Invitrogen, CA, USA) were used to prepare transfecting complexes in accordance with manufacturer protocols. For a vector-based RNAi approach, a double-stranded short hairpin RNA (shRNA) was cloned into the Lentiviral vector (GV248, hU6-MCS-Ubiquitin-EGFP-I RES-puromycin) by Genechem Corporation (SHANGHAI). On the basis of siRNA sequences, the particular sequence of shRNA was same to siRNA 2 sequence. The vector was also inserted with scramble sequence for control. Vectors either bought or constructed were verified through sequencing and then added with Lipofectamine 2000 (Invitrogen Life Technologies, CA, USA) to transfect EC 9706 and Eca 109 cells in accordance with manufacturer's instruction and then fluorescence microscope was utilized to monitor the expressing status of EGFP. Cells were collected 48 and 72 hours after being transfected to evaluate the mRNA and protein levels using qRT-PCR and western-blotting separately. Cells in negative control group were grown under normal conditions. All transfecting experiments were conducted for three times.

### RNA extraction and RT-PCR

Trizol reagent (Invitrogen Life Technologies, CA, USA) was utilized to isolate total RNA in accordance with the guideline of manufacturer. A NanoDrop ND 1000 was used to determine the integrality and purity of isolated RNA. DNase I was added into RNA samples to remove genome DNA contaminate in accordance with the manufacturer's instructions (TaKaRa, Dalian, China) and prime SCRIPTTM RT-PCR kit (TaKaRa, Dalian, China) was subsequently utilized to perform reverse transcript- tion with extracted RNA. IQ5 system (Bio-Rad, USA) was used to conduct qRT-PCR assays utilizing SYBR Select Master Mix (Boster, Wuhan, China) in accordance with manufacturer's instruction. After being normalized by β-actin internalcontrol, the relative expressions of the frozen tissues (human and mice) were measured by means of standard curves. The forward primer of qRT-PCR was 5′-TCCCATTTGTATCC CCTGAT-3′; the reverse primer was 5′-ATTCCCAGCAACA CTTCCAC-3′. Relative gene expressions of the ESCC both Eca 109 and EC 9706 cell lines were measured by means of comparative delta-delta CT method (2-ΔΔCt). And supplementary data 3 is the PCR primer imformation of target genes in the Figure 7D-7H. All experiments were conducted independently for three times. The PCR reacting conditions of reactive solutions which contained Thsd7a or β-actin were as follows: 95°C for three minutes, 95°C for ten seconds through forty cycles and 59°C (Thsd7a) and 58.5°C (β-actin) for thirty seconds.

### Protein isolation and western blotting

The lysing buffer of radio-immumoprecipitation assay (RIPA) was used to process cells at 72h after transfection. And the bicinchoninic acid protein quantitation assay (Bioteke, Beijing, China) was used to determine the concentration of protein. Six percent of SDS-PAGE gel was used to separate 80μg protein of each sample and the gel pieces were then transferred onto PVDF membranes (PVDF, Millipore, Bedford, USA) which were subsequently incubated and shaken in 5% skimmed milk under ambient temperature for one hour. Goat polyclonal first antibody against Thsd7a (1:1000 dilution; Santa Cruz Biotechnolgy) and rabbit polyclonal antibody against β-actin (1:1000 dilution; Boster, Wuhan, China) were added the whole night to hybridoma at 4°C. Blots were washed in PBS-Tween three times, after which the second antibody (Invitrogen Life Technologies) was added under ambient temperature for two hours and the bands were visualized using Western Breeze Kit (WB7105, Invitrogen). The quantification of bands and the normalization of β-actin intensity were conducted with Quantity One software (Bio-Rad Laboratories, CA).

### Cell proliferation assay

EC 9706 and Eca 109 cells were inoculated onto a 96-well plate and cultured overnight with an initial density of 2*103/well. At 0, 24, 48, 72, as well as 96 hours after transfection, 20μl (5mg/mL) of Methylthiazolyl blue tetrazolium (MTT; Genview, JT343) was used to treat cells in given wells. Four hours after that, dimethylsulfoxide (DMSO, 100μl/well) was used to dissolve MTT formazan precipitates in a shaker. Then the absorbing data at 490nm were read in a multiwall plate reader (Tecan infinite, M2009PR). All experiments were performed three times independently.

### Analyzing variations in apoptosis and cell cycle with flow cytometry

Flow cytometry (FCM) was used to analyze apoptosis and cell cycle. In the analysis of cell cycle, cells were seeded in six-well culture plates at a density of 3×105 cells/well, then transfected after 24 hours followed by incubation at 37°C in 5%CO_2_ for 48 hours. Harvest cells were fixed with 75% chilled alcohol and preserved under the temperature of −20°C over night. Phosphate buffer saline (PBS) was used to wash cells and 500μl PBS which contained RNase (50μg/mL) and 0.1%Triton X-100 was added into tubes to incubate cells under the temperature of 37°C for one hour. After that, 5μl of propidium iodide (PI, 1mg/mL) was added into each tube for 30 minutes of incubation on ice away from light. Subsequently, FCM (BD FACS Aria III) was used to detect cells which passed through a 200-mesh filter with the aim of counting and comparing cells in G0/G1, S as well as G2/M stages. For cell apoptotic analysis, an Annexin V-FITC apoptosis detection kit was used (Invitrogen), and Annexin V-FITC staining was performed according to instructions provided by the manufacturer. Cells were washed twice with cold PBS and added with 5μL annexin V and 100μL binding buffer totally, then added with 0.4μL of PI (10mg/mL) and incubated on ice away from light for 5 minutes. The examination with FCM was conducted within 30 minutes and all tests were conducted independently for three times.

### Cell would healing assay

Eca 109 and EC 9706 cells (5*105/well) were inoculated in six well plates and cultured with RPMI-1640 medium without serum. The monolayer cells were scraped in the center of the plate with a 10μl sterilized pipette tip to form a linear wound. Then siRNA was used to transfect the cells which were subsequently cultured under the conditions of 37°C and 5%CO_2_. Photos of cells in different groups were taken under an optical microscope at the time point of 0h, 24, 48h as well as 72h after the cells were transfected.

### Transwell migration and invasion assays

This experiment adopted the Transwell chamber in 8 μm/well from Corning, Inc. (Corning, NY, USA) to evaluate the metastasis and invasion ability of tumor cells. For the metastasis and invasion assays, esophageal cancer cells were seeded and transfected transitorily with siRNA and control RNA. 100 ml of serum-free media was used to culture the cells in the upper chamber. However, the lower chamber included 600 ml culture media containing 10% calf serum. After culturing for 10 h at 37°C, cells were separated from the membrane surface through a cotton swab. Subsequently, filters were fixed with methanol for 20 min and dyed *via* Giemsa for 30 min. The migrated cells were counted then. Each well counted 5 random fields (Nikon ECLIPSE TS100), and then the mean value was adopted. The membrane in the upper chamber of the transwell was coated with 100 ml of a 1mg/ml solution of Matrigel (BD, USA) in advance.

### Xenografted athymic nude mice model

The protocol of animal experiment in this study received approval from Animal Ethnics Committee of First Affiliated Hospital of Xinjiang Medical University. Eca 109-xenograted mouse model was constructed with the aim of evaluating the oncogenic capacity of Thsd7a *in vivo*. Eca 109 cells were implanted into fifteen BALB/c-nude female mice (weight 14-16g) which were divided into three groups at the age of five weeks. Experimental mice were kept in sterile environment under the cycle of twelve hours of light and twelve hours of night. Lentiviral-shRNA and control vector were transfected into Eca 109 cells which were subsequently sorted by FCM for purification and injected subcutaneously into the left side of naked mice. Euthanasia was conducted on all tested mice to collect and then weight the generated tumors. The following formula was used to calculate the volumes of tumors (TV) each 3 days through 4 weeks: TV(mm3) = length*width*width/2 [21].

### Microarray processing and analysis

Total RNA were extracted from normal control (*n* = 3) and lentivirus infected Eca 109 (*n* = 3) by Trizol regents. And then we used NanoDrop 2000 and Agilent Bioanalyzer 2100 to assess the quality and quantity of the total RNA. In accordance with the manufacturer's instructions, we used the Affymetrix human GeneChipprimeview chip to determine the gene expression profile. Further, by using the GeneChip 3′IVT Expression Kit, we completed reverse transcription, template conversion of double-stranded DNA, and synthesis of mRNA and labeled *in vitro* transcription. We made Chip hybridization, washing and staining by GeneChip Hybridization Wash and Stain Kit. And using the GeneChip Scanner 3000 to scan and produce the array data. We selected the significant difference in the two groups according to the standards of *P* < 0.05 and absolute fold change > 2. IPA is an integrated online integration analysis software (www.Ingenuity.com). Rely on the IPA, we performed Pathway enrichment analysis, and drawed Pathway control network and gene interaction network diagram. And supplementary data 2 is the description of IPA in the analysis of signal pathways and molecular network.

### Statistical analysis

Statistical analyses were determined using SPSS Version 21 (SPCC Inc., Chicago, IL). Fish's exact or Person chi-square tests were applied for evaluating the correlation between the expressing quantity of Thsd7a and characteristics of pathology. Consecutive data were processed using Student's *t* test of independent samples. One way ANOVA and Student's *t* test were used to calculate discrepancies between groups. Moreover, the remarkable differences in prognosis between patients were analyzed using K-M survival curve on the basis of log-rank test and Cox's proportional hazards regression model. Values were expressed as Means ± SD. P values were two-sided, and the significance level was *P* < 0.05.

## RESULTS

### The abundance of Thsd7a in ESCC tissues remarkably exceeded that of control

**Figure 1:**
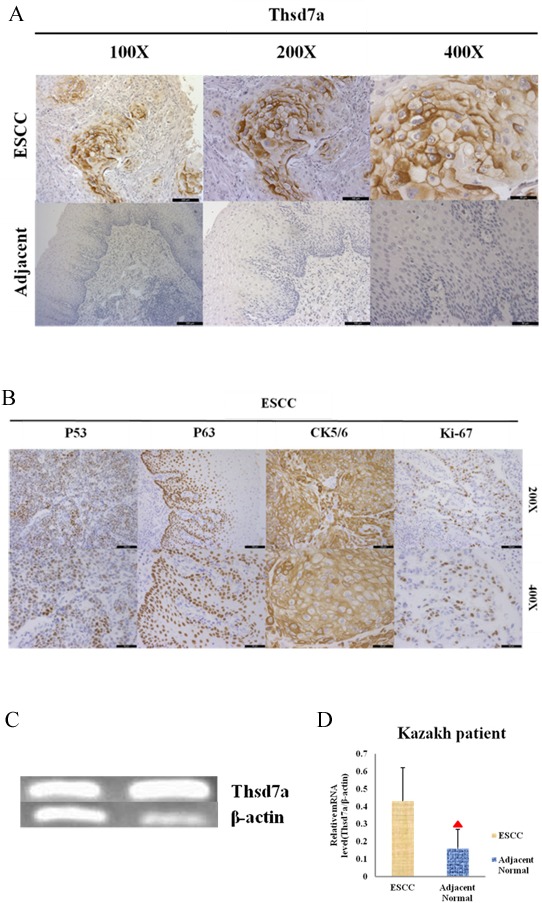
Immunohistochemical staining and RT-PCR for Thsd7a in ESCC and adjacent normal tissue of Kazakh patients **A.**, Positive and Negative staining in ESCC and paired adjacent normal tissue, separately. **B.**, Positive staining in ESCC for P53, P63, CK5/6, Ki-67. **C.**, Quantification of Thsd7a and β-actin mRNA in ESCC as standard product. **D.**, Relative expression of Thsd7a in ESCC and adjacent normal tissue of Kazakh patients. ▲ Compared with ESCC, P < 0.05.

Antibodies were tested on formalin-fixed, paraffin-embedded, adjacent normal tissues and ESCC. IHC was conducted with the aim of examining the expressing and localizing conditions of Thsd7a in ESCC from Kazakh patients. According to Figure 1A and Figure 6D, only nuclei and cytoplasm exhibited positive in immunostainings. In addition, we performed RT-PCR by 41 pairs frozen tissues of Kazakh patients. Both IHC and RT-PCR showed that the expressing quantity of Thsd7a in ESCC tissue remarkably exceeded that of surrounding normal tissue (*P* < 0.01, Table 1, Figure 1C,1D). In order to compare the correlation between Thsd7a and common markers of ESCC immunohistochemistry, we performed 4 other indicators of the same patient group (P53, P63, CK5/6, and Ki-67; Figure 1B). The positive rates and expression localization of these 4 indexes were consistent with previous studies [25–29], while the positive rate of Thsd7a for ESCC and adjacent normal tissue was 70.1% and 18.2%, respectively (Table 1). And the expression of these indicators, suggesting that the cancer of these patients had the characteristic of active proliferation and highly malignant degree of ESCC. And the coincidence expression rate of the 5 indicators was 55.6% (Table 1).

**Table 1:** Clinicopathological significances of Thsd7a expression in 117 Kazakh patients

Characteristics	n	Thsd7a expression		X^2^	P^a^
		Positive(%)	Negative(%)		
Specimen				62.461	<0.01
Adjacent normal tissue	111	20(18.0)	91(82.0)		
ESCC	117	82(70.1)	35(29.9)		
Sex				1.892	0.17
Female	30	24(80.0)	6(20.0)		
Male	87	58(66.7)	29(33.3)		
Age(y)				0.09	0.76
>60	61	42(68.9)	19(31.1)		
≤60	56	40(71.4)	16(28.6)		
Family history				0.06	0.82
Negative	82	58(70.7)	24(29.3)		
Positive	35	24(68.6)	11(31.4)		
Clinical stage				6.35	0.04*
0-I	5	5(100.0)	0(0.0)		
II	89	57(62.4)	32(26.6)		
III	23	20(87.0)	3(13.0)		
Tumor location				0.64	0.73
Lower	47	31(66.0)	16(34.0)		
Middle	59	43(72.9)	16(27.1)		
Upper	11	8(72.7)	3(27.3)		
T classification				2.35	0.59*
Tis-T1	5	5(100.0)	0(0.0)		
T2	49	34(69.4)	15(30.6)		
T3	62	42(67.7)	20(32.3)		
T4	1	1(100.0)	0(0.0)		
N classification				0.92	0.34
N0	76	51(67.1)	25(32.9)		
N1-3	41	31(75.6)	10(24.4)		
Differentiation				14.69	0.001
Well	26	24(92.3)	2(7.7)		
Moderately	64	46(71.9)	18(28.1)		
Poorly	27	12(44.4)	15(55.6)		
Tumor volume(cm^3^)				0.21	0.91
≤10	47	34(72.3)	13(27.2)		
10-40	47	32(68.1)	15(31.9)		
>40	23	16(69.6)	7(30.4)		
Gross classification				5.29	0.12
Fungating type	12	10(83.3)	2(16.7)		
Ulcerative type	72	54(75.0)	18(25.0)		
Medullary type	27	15(55.6)	12(44.4)		
Constrictive type	4	2(50.0)	2(50.0)		
Vascular Invasion				0.01	0.95
Negative	94	66(70.2)	28(29.8)		
Positive	23	16(69.6)	7(30.4)		
Nerve Invasion				2.16	0.14
Negative	98	66(67.3)	32(32.7)		
Positive	19	16(84.2)	3(15.8)		

^a^ Bold type indicates statistical significance.

^*^ indicates the result of fish's exact test.

Abbreviations: ESCC, Esophageal squamous cell carcinoma; Family history, history of cancer within three generations of the lineal family; Clinical stage: according to the American Joint Committee on Cancer staging standard; Tumor volume: tumor length * width * width/2.

**Table 2:** Positive rates of five characteristics' expression in Kazakh patients by IHC

Characteristics	Localization	Positive rate of ESCC(%)	Groups of positive rate(%)			Positive coincidence rate(%)
			Weak	Moderate	Strong	
P53	nucleus	68.2	31.1	28.9	40	55.6
P63	nucleus	56.5	9.5	19.1	71.4	
CK5/6	cytoplasm+membrane	84.5	9.8	27.5	54.9	
Ki-67	nucleus	100	24	60	16	
Thsd7a	cytoplasm+membrane	70.1	97	3	0	

### The expressing quantity of Thsd7a was remarkably related to the clinical stages and differentiation degrees of ESCC

Using the Chi-squared test, we found some significant differences in the demographic characteristics between the positive and negative expression of Thsd7a from Kazakh patients with ESCC. Specifically, there was significant correlation between Thsd7a expression and Clinical stage (*P* = 0.04) and Differentiation (*P* < 0.01). In other words, the later Clinical stage and the higher degree of tumor differentiation were, the higher expressing quantity of Thsd7a would be. Nevertheless, the expressing quantity of Thsd7a was not significantly associated with other clinical and pathological parameters which included tumor location, T/N classification, Tumor volume, Gross classification, vascular invasion, nerve invasion as well as demographic parameters (sex, age and tumor family history) in ESCC tissues (Table 1).

### Thsd7a expressing quantity was not related to prognosis

**Figure 2:**
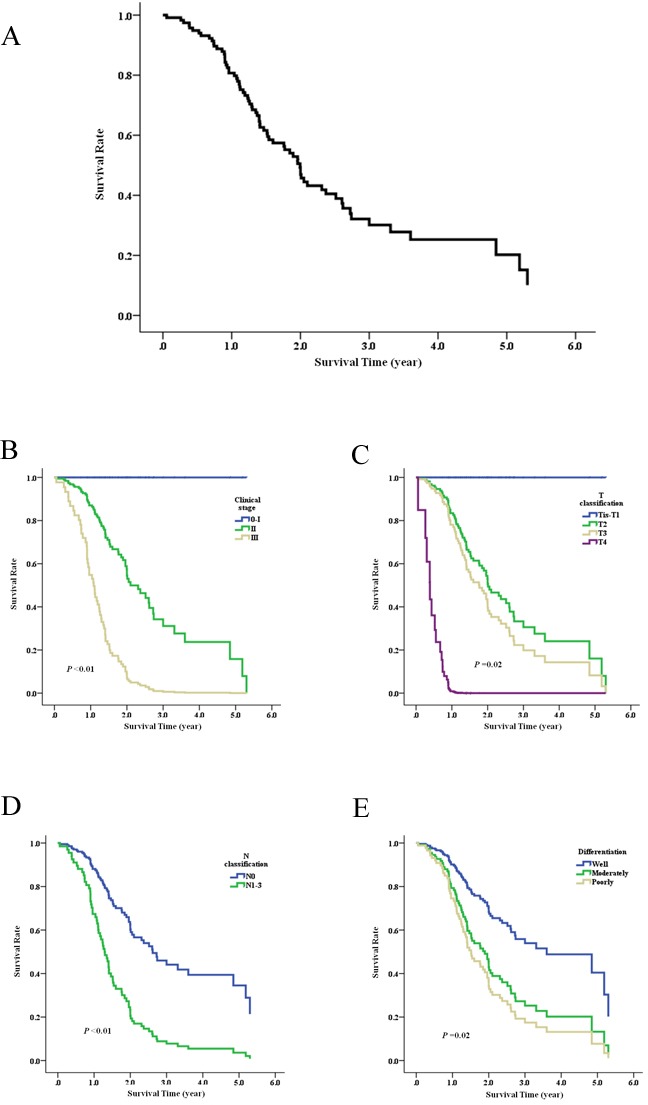
Survival analysis of ESCC *vs* Thsd7a expression evaluated using Kaplan-Meier curves **A.**, overall survival curve of Kaplan-Meier. **B.**-**E.**, survival curves of Clinical stage, T/N classification, and Differentiation, which were associated with prognosis analysised by Kaplan-Meier.

The correlation between prognosis for Kazakh patients and the expressing quantity of Thsd7a was analyzed by means of the construction of K-M survival curves and COX proportional regression analysis on the basis of prognosis records from 117 patients. As of May 15, 2016, the medium duration of follow-up was 1.9 years (range, 0.1 to 6.8 years). The 1, 3, 5 years overall survival rate of Kazakh patients was 80.7%, 32.1%, and 20.2%, separately (Figure 2A). The prognoses were not significantly variant between patients with positive and negative Thsd7a expressions (Table 3), while Kaplan-Meier univariate analysis showed that Clinical stage, T/N classification, and Differentiation were associated with prognosis of the Kazakh patients (Figure 2B-2E, Table 3). And COX multivariate analysis showed that Clinical stage, T/N classifica- tion were independent prognostic factors for the Kazakh patients (Table 3).

**Table 3:** Univariate and multivariate Cox analysis of ESCC in Kazakh patients, Xinjiang

Variables	Univariate		Multivariate^a^	
	HR(95%CI)	P^b^	HR(95%CI)	P^b^
ESCC		0.27		0.85
Negative	1		1	
Positive	0.75(0.46-1.24)		1.07(0.54-2.13)	
Sex		0.49		0.69
Female	1		1	
Male	1.23(0.68-2.21)		1.14(0.60-2.20)	
Age(y)		0.11		0.73
>60	1		1	
≤60	1.49(0.93-2.38)		0.90(0.50-1.63)	
Family history		0.58		0.75
Negative	1		1	
Positive	1.15(0.70-1.91)		1.11(0.59-2.09)	
Clinical stage		**<0.01**		-
0-I	1		-	
II	37063.31(0-2.70E55)		-	
III	160735.00(0-1.17E56)		-	
Tumor location		0.89		0.58
Lower	1		1	
Middle	1.22(0.54-2.77)		1.78(0.58-5.47)	
Upper	1.05(0.63-1.75)		1.05(0.57-1.92)	
T classification		**0.02**		0.03
Tis-T1	1		1	
T2	41458.33(0-2.80E58)		1.56(0.45-5.38)	
T3	56579.73(0-3.82E58)		1.82(0.54-6.09)	
T4	1080852.39(0-7.43E59)		14668.19(22.17-9706425.24)	
N classification		<0.01		**0.03**
N0	1		1	
N1-3	3.12(1.94-5.01)		2.23(1.10-4.51)	
Differentiation		**0.02**		0.71
Well	1		1	
Moderately	2.23(1.13-4.42)		1.37(0.65-2.86)	
Poorly	2.83(1.35-5.93)		1.35(0.53-3.46)	
Tumor volume(cm^3^)		0.07		0.68
≤10	1		1	
10-40	1.83(1.09-3.09)		1.26(0.65-2.45)	
>40	1.69(0.86-3.32)		0.90(0.36-2.24)	
Gross classification		0.36		0.85
Fungating type	1		1	
Ulcerative type	1.50(0.67-3.35)		1.39(0.58-3.35)	
Medullary type	1.34(0.54-3.31)		1.57(0.58-4.25)	
Constrictive type	3.05(0.88-10.52)		1.30(0.27-6.20)	
Vascular Invasion		0.06		0.78
Negative	1		1	
Positive	1.73(0.97-3.09)		0.89(0.38-2.06)	
Nerve Invasion		0.17		0.44
Negative	1		1	
Positive	1.58(0.82-3.04)		1.43(0.58-3.48)	

^a^ Adjusted by Cox proportional hazard regression model including all factors, as categorized in the Table.

^b^ Bold type indicates statistical significance.

Abbreviations: ESCC, Esophageal squamous cell carcinoma; Family history, history of cancer within three generations of the lineal family; Clinical stage: according to the American Joint Committee on Cancer staging standard; Tumor volume: tumor length * width * width /2.

### Transfecting cells with siRNA for knocking out Thsd7a

**Figure 3:**
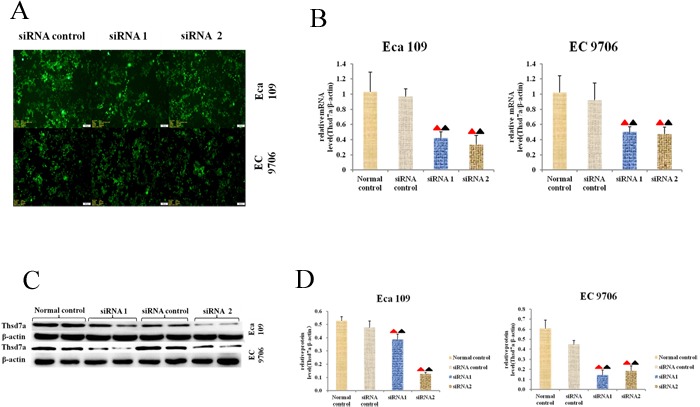
Expression of Thsd7a was knocked out with specific siRNA1 and siRNA2 **A.**, Morphology of transfected Eca 109 and EC 9706 cells under microscopy (magnifycation 200X). **B.**, The relative mRNA expression of Thsd7a was displayed, which normalized by β-actin. **C.**, The level of Thsd7a protein detected by Western blotting after transfection for 72h. **D.**, The relative protein expression of Thsd7a was displayed, which normalized by β-actin. ▲ Compared with Normal control, P < 0.05. ▲ Compared with siRNA control, P < 0.05.

The effect of Thsd7a on cells *in vitro* was explored by means of transiently knocking out Thsd7a through transfecting Eca 109 and EC 9706 cells with anti-Thsd7a siRNA. According to Figure 3A-3F, the expressing quantities of Thsd7a in Eca 109 and EC 9706 cells were reduced by siRNA. The relative transcribing and expressing quantities of Thsd7a (Thsd7a/β-actin) in the group treated with siRNA were remarkably inferior to those in the siRNA control and normal control group (*P* < 0.05; Figure 3B-3F), which suggested that siRNA could knock out Thsd7a. Because the interference efficiency of siRNA1 and siRNA2 was similar, so the follow-up cytology experiments were set up two interference groups (siRNA1 and siRNA2).

### The proliferating activity of EC 9706 and Eca109 cell lines was inhibited by the knocking out of Thsd7a

**Figure 4:**
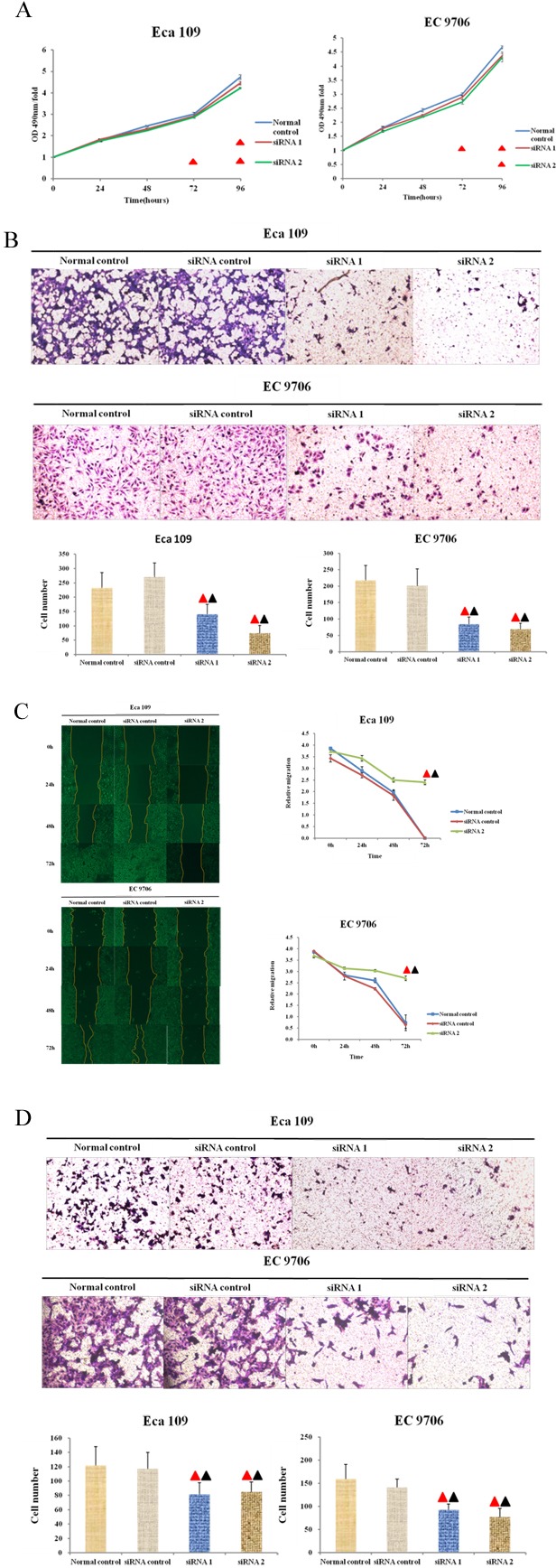
Thsd7a promotes proliferation, migration, and invasion in ESCC cell lines Eca 109 and EC 9706 **A.**, MTT assay of Eca 109 and EC 9706 cells at 0 to 96 hours after transfection with Thsd7a-siRNA and Normal control. **B.**, Migration of Eca 109 and EC 9706 cells by Transwell assay. **C.**, Wound healing assay of Eca 109 and EC 9706 cells at 0-72 hours after transfection with Thsd7a-siRNA and Normal control. **D.**, Invasion of Eca 109 and EC 9706 cells by Transwell assay. ▲ Compared with Normal control, P < 0.05. ▲ Compared with siRNA control, P < 0.05.

MTT was conducted to determine the changes of in Eca 109 and EC 9706 cells in proliferating process after being transfected using anti-Thsd7a siRNA. According to Figure 4A, three groups were not different from one another significantly in statistics at zero and 24 hours (*P* > 0.05). Nevertheless, the proliferating activity of Eca109 was notably inhibited after which was transfected with siRNA for 48 hours (siRNA1 for 72 hours) in comparison to control group (*P* < 0.05), although the proliferating activity of EC 9706 was under significant inhibition after which was transfected with siRNA1 for 48 hours (siRNA2 for 72 hours) compared with the Normal control group (*P* < 0.05), which indicated that the proliferating activity of EC 9706 and Eca109 cell lines was under the suppression of knocking out Thsd7a (Figure 4A).

### The migrating activities of EC 9706 and Eca 109 cell lines were suppressed by the knocking out of Thsd7a

Transwell cell migration and wound-healing assays were conducted with the aim of determining the influence of Thsd7a on migrating activity of cells. Firstly, we performed a Transwell migration assay in 4groups (Normal control, siRNA control, siRNA1 and siRNA2). The results showed that cell migration abilities of Eca 109 and EC 9706 were inhibited after reduced expression of Thsd7a compared with control groups(Figure 4B). And the inhibiting ability of siRNA2 was stronger than that of siRNA1(Figure 4B). In addition, we also made a wound-healing test to prove migrating influence of siRNA2-Thsd7a. After 48 hours of transfection using anti-Thsd7a siRNA2, the scrapes of experimental groups including EC 9706 and Eca 109 cell lines were remarkably broader than those of control groups (siRNA-control and Normal control). The relative migration of the group transfected with anti-Thsd7a siRNA were significantly different in statistics from that of control group, which also suggested that the migrating activities of EC 9706 and Eca 109 cell lines were remarkably inhibited by knocking out Thsd7a (Figure 4C).

### The invading activities of EC 9706 and Eca109 cell lines were suppressed by knocking out Thsd7a

Transwell cell invasion assays were conducted to evaluate the influence of knocking down Thsd7a on the invading capacity of ESCC cell lines. In Figure 4D, the quantities of cells in 4 groups (Normal control, siRNA control, siRNA1, and siRNA2) passing through membranes coated with matrigel were displayed. It was revealed that the quantities of normal and siRNA control groups were notably superior to those of experimental groups which contained EC 9706 and Eca 109 cell lines transfected with siRNA (*P* < 0.05, Figure 4D), which indicated that the invading activities of EC 9706 and Eca 109 cell lines were suppressed by knocking out Thsd7a.

### Knocking out Thsd7a could induce the arrest of cell cycles in G0/G1 phase

**Figure 5:**
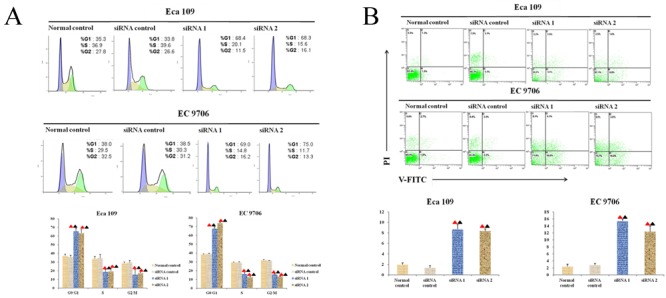
Thsd7a promotes apoptosis and cell cycle arrest at G1 phase after knockout **A.**, Cell cycle assay of Eca 109 and EC 9706 cells at 48 hours after transfection with Thsd7a-siRNA1, siRNA2, and siRNA-control. **B.**, Cell apoptosis assay of Eca 109 and EC 9706 cells at 48 hours after transfection with Thsd7a-siRNA1, siRNA2, and siRNA-control. ▲ Compared with Normal control, P < 0.05. ▲ Compared with siRNA control, P < 0.05.

Forty-eight hours after transfection, FCM was used to determine the point in cell cycles with the aim of observing the changes in cell cycles induced by transfecting siRNA into ESCC cell lines. According to Figure 5A, the ratio values of EC 9706 and Eca 109 cells stuck in G0/G1 phase in the experimental groups transfected with anti-Thsd7a siRNA (siRNA1 and siRNA2) were remarkably higher than those in control groups (*P* < 0.05), which indicated that knocking out Thsd7a could induce the arresting of cell cycles in G0/G1 phase.

### Apoptosis in EC 9706 and Eca 109 cell lines could be promoted by knocking out Thsd7a

FCM was also utilized to examine the apoptotic rates after 48 hours of transfection. It was revealed in Figure 5B that the apoptotic rates of EC 9706 and Eca 109 cell lines for the siRNA-Thsd7a groups (siRNA1 and siRNA2) were significantly increased compared with the control group (*P* < 0.05), suggesting that silencing of Thsd7a promotes apoptosis of Eca 109 and EC 9706 cells.

### Knockdown of Thsd7a could inhibit the oncogenesis in athymic naked mice with xenografts induced by Eca 109 cell lines

**Figure 6:**
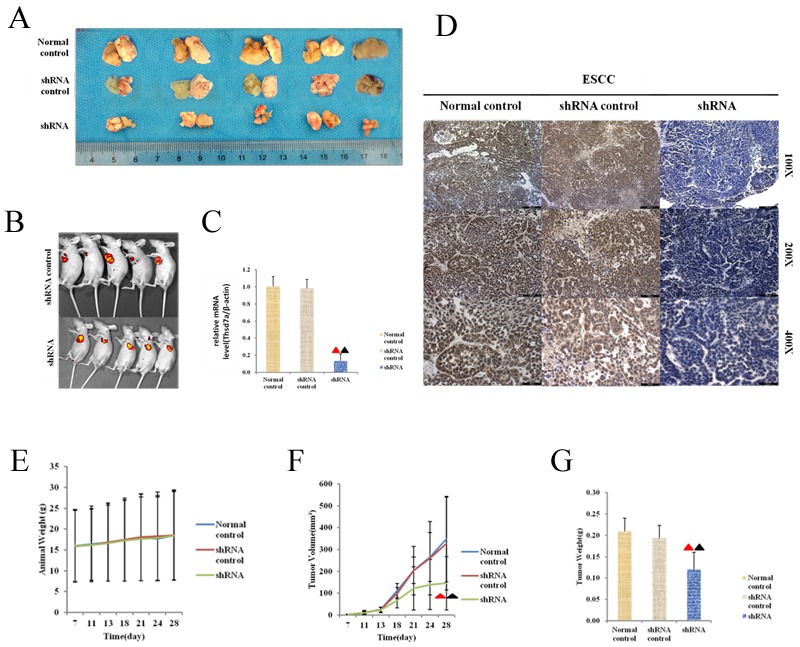
Effect of silencing expression of Thsd7a in tumorigenesis of athyhmic nude mice xenografted with Eca109 **A.**, Morphology of FCM sorted Eca 109 cells whose basal level of Thsd7a was stably knocked down using lentiviral recombinant vector produced smaller tumor lesions, compared with Normal and shRNA control. **B.**, The vivo image of nude mice injected with shRNA control and shRNA. **C.**, The relative mRNA level of tumor body of nude mice treated by shRNA. **D.**, Immunohistochemical staining of tumor body of nude mice treated by shRNA. **E.**, Quantitation of animal weight for tumorigenesis in athymic nude mice. **F.**, Quantitation of tumor volume for tumorigenesis in athymic nude mice. **G.**, Quantitation of tumor weight for tumorigenesis in female athymic nude mice. ▲ Compared with Normal control, P < 0.05. ▲ Compared with shRNA control, P < 0.05.

The influence of Thsd7a on oncogenesis *in vivo* was verified by means of athymic naked mice model. First, lentivirus vectors were used to transfect Eca 109 cells in order to steadily knock down the innate Thsd7a level based on its background level. And consequently, a transgenic Eca 109 cell line was obtained. The sequence of shRNA was consistent with siRNA2, and the interference efficiency of shRNA was more than 85%. The steadily transgenic Eca 109 cell line was sorted by FCM using puromycin to maximize the purity. Subsequently, control and sorted transgenic Eca 109 cells were inoculated in subcutaneous tissues of athymic naked mice. The weight of nude mice and tumor volume were measured once every 3-4 days from 7 days after the injection of Eca 109 cells. Four weeks after injection, euthanasia was performed on experimental mice. The scale and volume of tumorous tissue were determined and Thsd7a expression was tested by RT-PCR and IHC (Figure 6A-6D). And the vivo image (Caliper Life Sciences, MJ02PQHZ, Hopkinton, USA) of nude mice was carried out under the anesthesia of the Hydrate hydrate (30μL/10g) in the Figure 6B. It was found that knockdown of Thsd7a could suppress the oncogenesis in athymic naked mice with exnografts induced by Eca 109 cell lines (Figure 6F, 6G), which casually confirmed the oncogenic effect of Thsd7a on Eca 109 cell lines *in vitro*. Moreover, differentiat- ing activity was considered as remarkably related to Thsd7a *in vivo*, which accorded with the oncogenic property of well differentiation Eca 109 cells *in vivo* in nude mice and vitro. However, there is no significantly different effect on the total mice weight (Figure 6E).

### A variety of essential signaling pathways which participated in tumor progression were disrupted by knocking down Thsd7a

**Figure 7:**
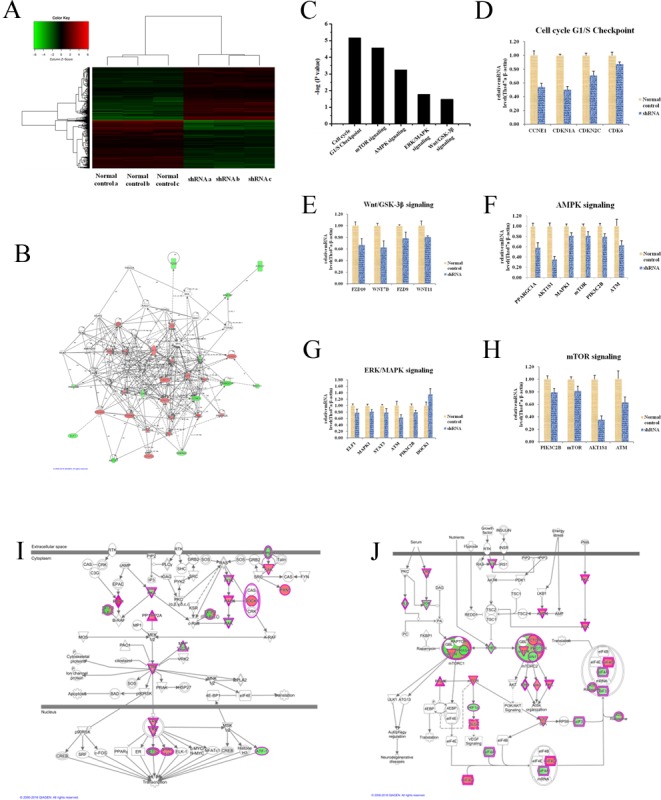
Widespread changes of gene expressions in Eca109 cell lines with Thsd7a knockdown by microarray **A.**, Heatmap representation of 20000 genes through RefSeq or via UniGene annotation showed significant differential expressions in ESCC Eca 109 cell lines infected with lentivirus expressing either shRNA and Normal control under the criteria P < 0.05 and the absolute value of fold change > 2. **B.**, Networks were constructed between Thsd7a and genes involved in IPA analysis. **C.**, Functional pathway enrichment of differential genes was analyzed based on IPA dababases. **D.**, **E.**, **F.**, **G.**, **H.** Relative mRNA expression of genes in the five enrichment pathways (P values of all showed genes < 0.05), Cell cycle G1/S Checkpoint, Wnt/GSD-3β signaling, AMPK signaling, ERK/MAPK signaling, and mTOR signaling. I, J, Functional interaction networks between Thsd7a and genes involved in ERK/MAPK and mTOR signaling.

According to above-mentioned results, Thsd7a was essential to tumorous development of ESCC cells and Kazakh patients. However, mechanisms underlying Thsd7a-mediated ESCC development and its downstream pathways are still unrevealed. Consequently microarray platform (3 Normal control chips Vs 3 shRNA Thsd7a chips) was used to examine Eca 109 cell line after infection with lentivirus which could express Thsd7a-shRNA with the aim of identifying genes exhibiting remarkable differential expressing status (*P* < 0.05 and absolute fold change (FCAbsolute) > 2), including upregulated genes and downregulated genes (Figure 7A). Based on the result of chips, the gene interaction networks of Thsd7a were shown by Ingenuity Pathway Analysis (IPA) in Figure 7B. Then KEGG pathway analysis was enriched in five pathways significantly, based on a *P* < 0.001 threshold. These pathways, including mTOR Signaling, Cell Cycle G1/S Checkpoint, Wnt Signaling, AMPK signaling, and ERK/MAPK Signaling, were all critical for cancer development and progression (Figure 7C). We performed RT-PCR validation of the major genes in these pathways. The Figure 7D-7H, showed the significant difference genes in these pathway through the comparison of Normal control and shRNA groups. To the downstream genes which included in both literatures and chips, but no significant difference results for chip and RT-PCR, we showed them in the supplemental material 4. Functional interaction network analysis was further employed to investigate the relationship between Thsd7a and genes involved in ERK/MAPK signaling and mTOR Signaling based on the analysis of chips, which were our two most interested pathways (Figure 7I, 7J).

## DISCUSSION

The overexpression of Thsd7a in ESCC and remarkable differences in the expressing quantity of Thsd7a between adjacent normal tissue and ESCC (mRNA and protein level) were discovered in this study. And it was correlated with Clinical stage and differentiation in ESCC of Kazakh patients, Xinjiang. While no significant difference was discovered between Thsd7a expressing quantity and other clinical- pathological parameters which included tumor location, TNM stage, tumor volume, gross classification, vascular invasion, nerve invasion, and prognosis. The proliferating, migrating as well as invading activities were accelerated by Thsd7a among ESCC cells.

It was proposed that Thsd7a protein actively participated in Osteoporosis, Membranous Nephropathy and Obesity [8–12], and has significantly genetic variations in some exons. Consequently, an overwhelming majority of studies on the role of Thsd7a in these diseases were devoted into the relationship between genetic variations of Thsd7a and its clinicopathological phenotypes, affecting the features of clinical and disease progression [13–16, 22]. Comparatively, the expressing and transcribing quantity, gene function, mechanism and pathway were still unrevealed in cancer, especially in ESCC. This is the first report of Thsd7a has a certain role in the occurrence and development of ESCC. In addition, this is the first report to show an association between Thsd7a staining and clinicopathological features in ESCC of Kazakh patients in Xinjiang, China.

In the present work, we found that there were significant correlations between Thsd7a expression and several clinicopathological factors, such as clinical stage, and differentiation. That is to say, Thsd7a expression is higher for the cancer tissues of worse stage and better differentiation. It is well known that the degree of cell differentiation suggests the malignancy of the tumor. And highly differentiated cancer means a lower degree of malignancy. Although Thsd7a could promote cancer, it may be associated with high differentiation. We speculate that the possible reason is that Thsd7a could promote proliferation and migration, but it may have a weak effect on cell morphology and differentiation ability. On the other side, because the sample size is limited and stratified, the conclusion based on the statistical method could only be used as a cue. The corresponding results also require more research and larger sample size to validate. Thsd7a is mainly expressed in cell membrane, which is entirely concordant with the previous findings in Membranous Nephropathy [23, 24], and there is a little expression in the cytoplasm, which was never sported before. We used P53, P63, CK5/6, Ki-67 as positive control, which showed that the tumor tissues collected were higher malignant ESCC, and the positive rates of the markers were similar with the early reports [25–29]. The positive rate (70.1%) and positive coincidence rate (55.6%) showed that Thsd7a had higher expression in ESCC of Kazakh patients, Xinjiang.

The proliferating, migrating as well as invading capacities of EC 9706 and Eca 109 cell lines of ESCC were prohibited by knocking out Thsd7a using particular siRNA1 and siRNA2. And the result of athymic nude mice model assay was similar with the cell level's in the ability of proliferation. Those data also highly confirmed the results of previous studies of Meng Wei et al [16, 30]. However, the effect of proliferation *in vitro* experiment was weaker than that *in vivo*. The possible reason is that the interference (siRNA) of the gene is not one hundred percent (also weaker than shRNA). So, it may cause many cells have not been completely blocked in the G1 phase, resulting in weakly effects on cell growth *in vitro*. In addition, promoted enhanced those abilities of ESCC cells, were important signs of malignant tumors [31]. It was convinced by evidences from the results that Thsd7a was critical to the progressing activity of tumors, which just confirmed the prediction of Chieh Huei Wang et al [5]. Moreover, the variations and apoptosis induced by knocking out Thsd7a, the rate of apoptotic cells as well as the ratio of cells arrested in G1 phase were elevated notably. These changes in cell cycle and apoptosis are general phenomenon in the process of tumor progression [32].

It was noteworthy that the correlations between Thsd7a and metastasis *in vitro* and *in vivo* were still controversial. In spite of few samples *in vivo*, it was revealed that Thsd7a was not associated with lymph node metastasis. However, according to the results of correlation assays in which Thsd7a of Eca 109 and EC 9706 cell lines were knocked out using siRNA1 and siRNA2, Thsd7a was capable of promoting cell invasion and migration, indicating the essentiality of Thsd7a to motion capacity *in vitro*, which agrees with previous non-tumor study of Eichmann A's [17]. The contradiction between experimental results *in vitro* and *in vivo* was probably explained in several ways in which one probability was the limited sample size of the Kazakh patients we collected, although we had gathered as much as possible. Secondly, considering the differences of human (minority: Kazakh, vivo) and cell line (Eca 109 and EC 9706, vitro), tumor (this study) and non-tumor (other researches), there might be some biased results, which need a large panel of ESCC patients and cell lines with the endogenous amount of Thsd7a.

In summary, the essential functions of Thsd7a in the developing and progressing activity of ESCC were verified using human ESCC cell lines of Eca 109 and EC 9706 as well as resection specimens through vitro and vivo experiments. However, relevant studies on the molecular mechanisms of the function of hsd7a in ESCC were still rare. With the aim of obtaining a deeper insight into the principles of Thsd7a-induced ESCC development and migration, Thsd7a of Eca 109 cells were knocked down and microarray analyses were performed on these cells. The results indicated that the expressing quantities of thousands of genes exhibited remarkable differences. In addition, gene signatures were analyzed with functional pathway analysis, and a variety of pathways associated with cancer development as well as functional interaction network which interested us were also demonstrated. For instance, cell cycle G1/S checkpoint as an essential factor for many cellular processes, for example the regulation of cell cycles, was associated with the development of many cancers and considered as a possible target for treating cancer [33]. Strikingly, the data of cell cycle G1/S checkpoint were complete agreement with the previous results of cell cycle assay, which further verified that the cell cycle was blocked in the G1 phase after the knockout of Thsd7a. The second significantly enriched pathway, mTOR signaling, the remarkable enriched pathways after Thsd7a was knocked down, was essential to the proliferating, migrating, and invading activity of tumors [34, 35]. Moreover, the same to the previous studies [30], Thsd7a had been confirmed that it was significantly related with ERK/MAPK signaling, which promotes the role of intermediary and amplified signal in the process of tumor invasion and metastasis [36, 37]. Other pathways, such as AMPK, and Wnt signaling, have been closely related to cancer development, too.

In addition, in view of these possible pathways, we performed RT-PCR validation of the major genes in the pathway. And the significant difference genes had been proved again by RT-PCR through the vitro assay on the comparison of Normal control and shRNA groups. Although some genes of previous studies was not proved the correlation with Thsd7a, such as TGF-β, we would perform more experiment to validate them.

The causal role of Thsd7a in the developing process of ESCC was testified by believable evidences from the results. It was also indicated by results of microarray analyses that Thsd7a was possibly an upstream factor which accelerated the developing and progressing activity of tumors. Moreover, it was revealed that Thsd7a could promote the proliferating, migrating as well as invading processes of tumors while the specific principle of tumorous progression was still unknown and would be uncovered in the future. Further studies on the molecular mechanism would expand our knowledge scope of the function of Thsd7a in tumorous developing process. In sum, the essentiality of Thsd7a in ESCC, especially for Kazakh patients was verified in this study which indicated that Thsd7a might be used as a biomarker for diagnosing ESCC and a target for treating ESCC of Kazakh patients, Xinjiang.

## SUPPLEMENTARY MATERIALS FIGURES AND TABLES


