# Supplementary material for: Expression, prognosis and functional role of Thsd7a in esophageal squamous cell carcinoma of Kazakh patients, Xinjiang
Source: Oncotarget. 2017 Apr 8;8(36):60539–57. doi: 10.18632/oncotarget.16966 (PMC5601160; doi:10.18632/oncotarget.16966)
Supplement: Supplementary file 1 [file oncotarget-08-60539-s001.pdf]

# Expression, prognosis and functional role of Thsd7a in esophageal squamous cell carcinoma of Kazakh patients, Xinjiang

## Supplementary Material

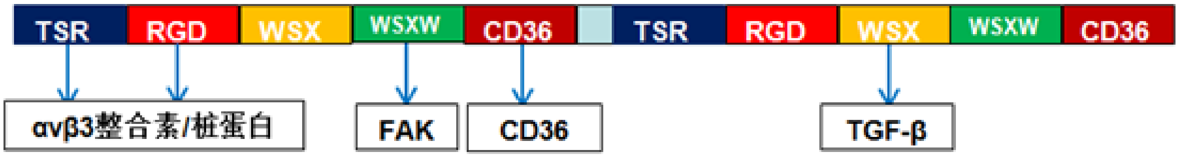


**Supplementary Data 1: The structure and combined target sketch map based on non-tumor researches**

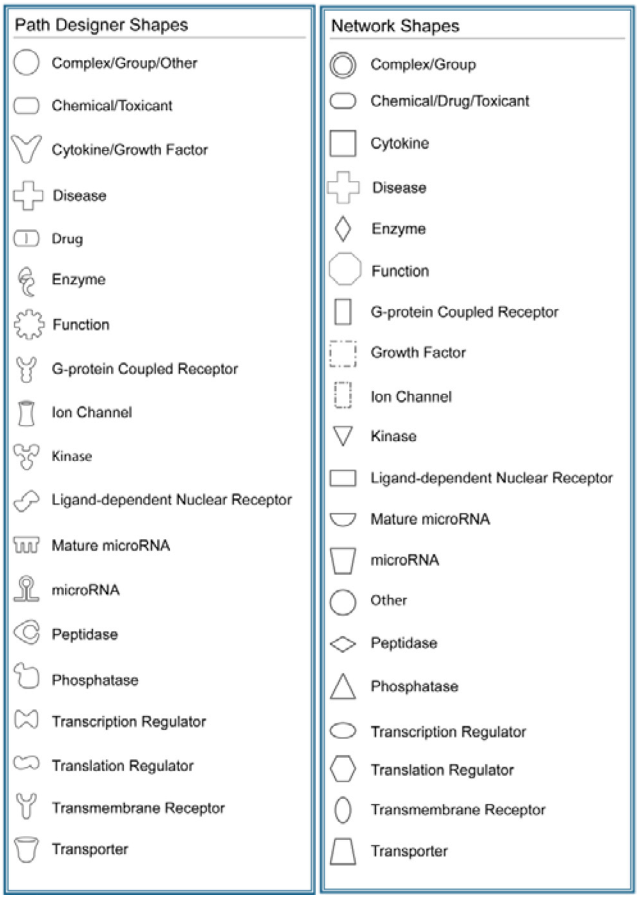

1 Molecular shape

**Relationship Labels**

| | |
|---|---|
| A | Activation |
| B | Binding |
| C | Causation/Leads to |
| CO | Correlation |
| CC | Chemical-Chemical interaction |
| CP | Chemical-Protein interaction |
| E | Expression (includes metabolism/ synthesis for chemicals) |
| EC | Enzyme Catalysis |
| I | Inhibition |
| L | Molecular Cleavage (includes degradation for Chemicals) |
| LO | Localization |
| M | Biochemical Modification |
| miT | microRNA Targeting |
| MB | Group/complex Membership |
| nTRR | Non-Targeting RNA-RNA Interaction |
| P | Phosphorylation/Dephosphorylation |
| PD | Protein-DNA binding |
| PP | Protein-Protein binding |
| PR | Protein-RNA binding |
| PY | Processing Yields |
| RB | Regulation of Binding |
| RE | Reaction |
| RR | RNA-RNA Binding |
| T | Transcription |
| TR | Translocation |
| UB | Ubiquitination |

2 Type and label of interaction relationship

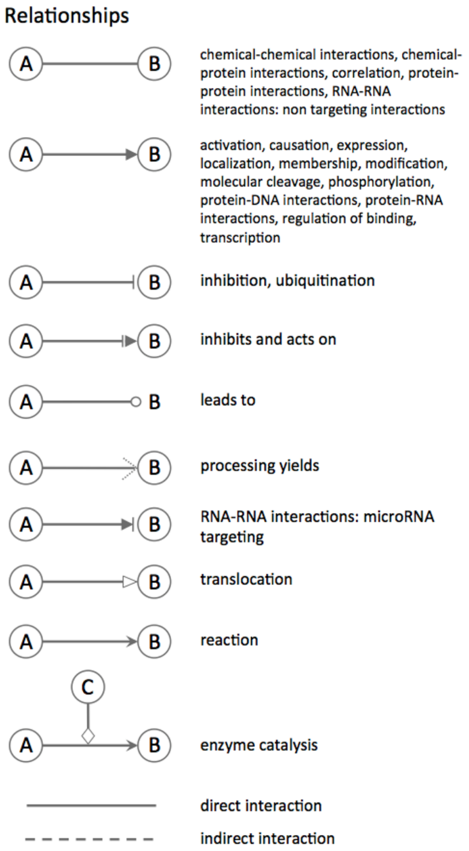


3 The relationship of correlated function

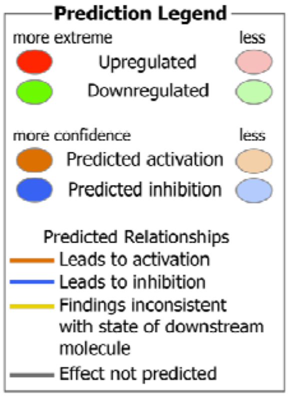


4 The prediction of molecular activation

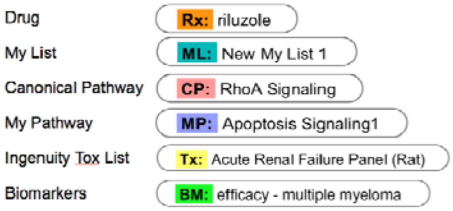


5 Molecular network and signaling pathways intersect notes

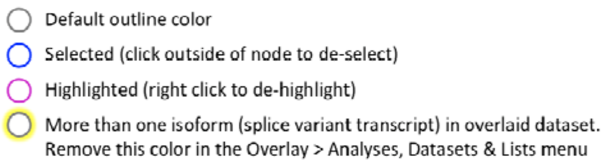


6 The molecular profile color in molecular network and signal pathway

**Supplementary Data 2: Description of IPA in the analysis of signal pathways and molecular network.**

**Supplementary Data 3: PCR primer informations of target genes.**

| Target gene | Forward primer | Reverse primer |
|---|---|---|
| Thsd7a | CATTCCACACCACAAGACAGTGAG | GAGACCCACGTCAGCTTTAGAACA |
| STAT3 | AGAAGGACATCAGCGGTAAG | CCTTGGGAATGTCAGGATAGAG |
| CDKN1A | CTGTCACTGTCTTGTACCCTTGT | AAATCTGTCATGCTGGTCTGC |
| mTOR | TCCGACCTTCTGCCTTCAC | ATTGCCTTCTGCCTCTTATGG |
| FZD8 | AGCGAAGGGACACTTGATGG | AATGGAGGAAAAGGGGCTGG |
| AKT1S1 | ATAATGGAGGGCTCTTTGTGA | GGCTGCCATCATCTGTACTCT |
| PIK3C2B | TTGTGCTTTGGGGAGCAGAG | GGACGCCATCTCTATGGTGG |
| FZD10 | AGAAGAGCCGGAGAAAACCG | TCCCGTGGTGAGTTTTCTGG |
| ELF1 | CTTCCCCAATCTACAGGAGCC | AAGCTTCTTGGCCTTCAAGTATT |
| ATM | CCACCAGAATCTCAAGGAATCAC | AGTAGCAGCCAAGGACACC |
| DOCK1 | AGTTCAACCCCTCCAGGGAA | ACCAAAATTTTCAGGACTTTGGAGT |
| CDK6 | CCTTAGCACAGCACCAC | GGGATTTCTCAGCCAGT |
| TGFB1 | ACAATTCCTGGCGATACCTC | TAAGGCGAAAGCCCTCAAT |
| WNT7B | TCCACTGGTGCTGCTTCG | GTCACGGGTGCTGTTCTGC |
| MAPK1 | TTACGACCCGAGTGACGA | CTGTATCCTGGCTGGAATCT |
| WNT11 | AGCGAGGACTCTGCTCAAGG | CCTTCTGTTCCTGGTGGCTTC |
| CDKN2C | AGACTGCTACTTAGAGGTGCTA | CAGGTTCCCTTCATTATCC |
| PPARGC1A | TCTGAGTCTGTATGGAGTGACAT | CCAAGTCGTTCACATCTAGTTCA |
| CCNE1 | AGGTTTCAGGGTATCAGTGGTGC | CTTTCTTTGCTCGGGCTTTGTCC |
| β-actin | ATGATGATATCGCCGCGCTC | TCGATGGGGTACTTCAGGG |

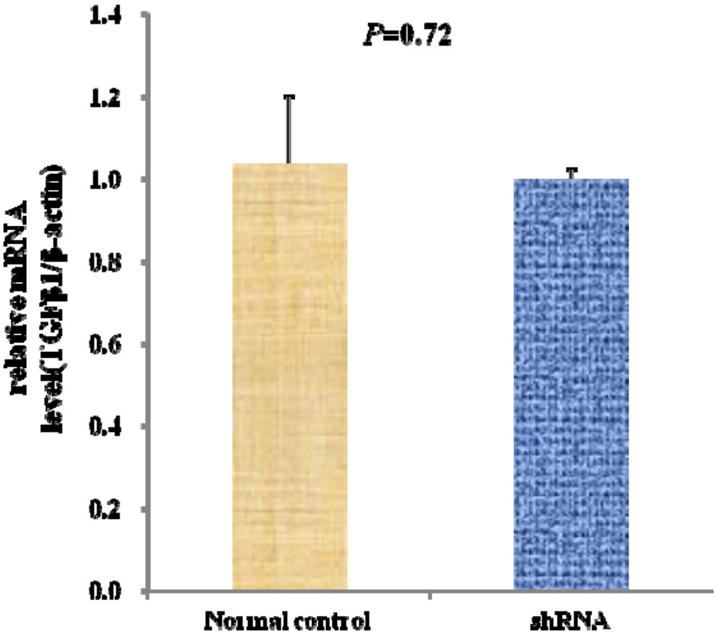


**Supplementary Data 4: Relative mRNA level of TGFβ1/β-actin.**
